# Characterization of the Tongue Worm, *Linguatula serrata* (*Pentastomida*), Identified from Hares (*Lepus europaeus*) in Romania

**DOI:** 10.3390/ijms241612927

**Published:** 2023-08-18

**Authors:** Beatrice Ana-Maria Jitea, Mirela Imre, Tiana Florea, Cătălin Bogdan Sîrbu, Iasmina Luca, Adrian Stancu, Alexandru Călin Cireșan, Gheorghe Dărăbuș

**Affiliations:** Faculty of Veterinary Medicine, University of Life Sciences “King Michael I” from Timisoara, Calea Aradului 119, 300645 Timisoara, Romania; mirela.imre@usvt.ro (M.I.); tijana.florea@usvt.ro (T.F.); catalin.sirbu@usvt.ro (C.B.S.); iasmina.luca@usvt.ro (I.L.); adrianstancu@usvt.ro (A.S.); alexandru.ciresan@usvt.ro (A.C.C.); gheorghedarabus@usvt.ro (G.D.)

**Keywords:** hares, *Linguatula serrata*, polymerase chain reaction, Romania, scanning electron microscopy

## Abstract

*Linguatula serrata* (Frölich, 1789) is a widespread parasite known as the tongue worm belonging to the family Linguatulidae. The adult form of the parasite is usually located in the upper respiratory tract of domestic and wild carnivores while the larval forms are located in the visceral organs of intermediate hosts (various herbivorous mammals). Twenty-four European brown hares (*Lepus europaeus*) were examined in this study, of which two were positive with *L. serrata* nymphs. The collected nymphs were examined morphologically using electron-microscopic analysis and molecularly by amplification of 18S rRNA and COX1 genes. Lung tissue samples were also collected and histopathological examination was performed. Histopathological examination revealed the following lesions: generalized inflammatory oedema, granulomas with necrosis, calcification and fibrosis in the bronchial tree. The results of molecular sequencing for *L. serrata* specimens collected from the European brown hares are deposited in GenBank. This study presents the first report on *Linguatula serrata* nymphs collected from *L. europaeus* in Romania, using molecular and morphological characterization simultaneously.

## 1. Introduction

The tongue worm, *Linguatula serrata* (Frölich, 1789), is a member of the subclass Pentastomida, order Porocephalida, family Linguatulidae. It is a cosmopolitan parasite whose adult form is located in the nasal cavities of carnivores (dogs, wolves or foxes), and larval forms are located in the lungs, liver and mesenteric lymph nodes of some mammals (cattle, sheep, goats, camels and rabbits) which act as intermediate hosts [1,2,3,4]. In addition to domestic and wild herbivores, Rajabloo et al. [5] (2015) also reported the Indian crested porcupine (*Histrix indica*) as an intermediate host.

Infection of definitive hosts is achieved by consumption of viscera and lymph nodes containing the nymph stage from intermediate hosts. Nymphs attach to the upper digestive tract or move rapidly from the stomach, reaching the nasopharynx where they pupate to the adult stage. Adults eliminate the eggs which are deposited on the mucus of the airways and later eliminated with secretions through sneezing or are swallowed and end up in the external environment with feces. They are ingested with forage by intermediate hosts, reach the small intestine, penetrate the intestinal wall, and settle in tissues, especially the lungs, liver and lymph nodes, where they develop to the nymph stage [6,7].

The prevalence of linguatulosis on a global scale varies depending on the host species. Numerous studies have indicated an overall prevalence of 21–58% in goats (South Africa and Iran) [8,9,10], 15% in sheep (Iran) [11], 7–19% in cattle (South Africa and Iran) [8,11], 8% in buffalo (South Africa) [8], 8% in donkeys [12] (Egypt) and 18.25% in camels (Iran) [13].

*Linguatula serrata* is of zoonotic significance, with several reports of infection in humans worldwide [14,15,16]. Infections have been reported in Asia, America, Europe, Russia, Africa and some Middle Eastern and Arab countries [6,17,18,19,20,21,22,23,24,25].

There are numerous reports of *L. serrata* infections in animals from Romania [26,27,28], including in dogs exported from Romania to the United Kingdom [29,30] and Germany [31]. Larval forms have apparently been found in mesenteric lymph nodes of sheep reared in traditional pastoral systems in different areas of Romania [32].

Despite examining a large number of wild animals in Romania, there have been no reports of *L. serrata* nymphs infecting hares. The aim of this study was to characterize, both morphologically and molecularly, *L. serrata* nymphs collected from the lungs of the European brown hares (*Lepus europaeus*) from Romania.

## 2. Results

**Figure 1:**
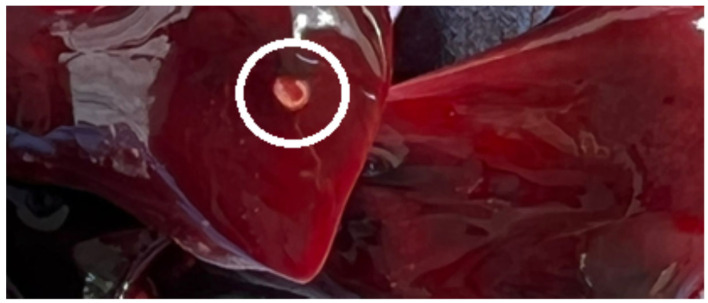
*Linguatula serrata* nymph encapsulated in the lung.

*Linguatula serrata* nymphs (*n* = 8) were observed in 2 out of 24 examined hares (Table 1). Five nymphs were found in the first hare while three were observed in the second one, both hares originating from Timis County (Traian Vuia and Ortisoara). All nymphs were located in the lung tissue and were encapsulated (Figure 1).

### 2.1. Morphology Results

Out of the eight recovered nymphs (Table 1), two were males and the other were females. This identification was based on morphological characteristics observed on the fifth row of abdominal rings, where male nymphs possess a genital pore or a gap between the two sensory papillae, and female nymphs have annular spines [4].

**Figure 2:**
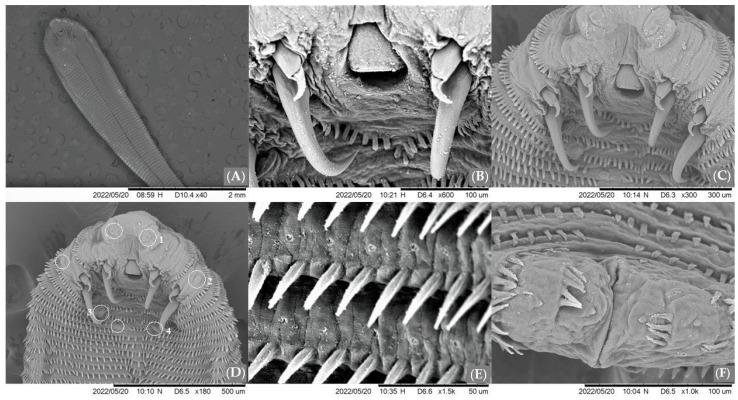
Electron microscopic scanning of *Linguatula serrata* female nymphs collected from hares in this study. (**A**)—Ventral part of the whole specimen. (**B**)—Enlarged view of the mouth. (**C**)—Enlarged view of the hooks. (**D**)—Anterior end with sensory papillae outlined and numbered to correspond to the description in the text. (**E**)—Enlarged view of annular spines and sensory pores. (**F**)—Posterior part of the female nymph.

Generally, female nymphs (Figure 2A) had a body length of 4.2 mm (3.0–5.5 mm) and a width of 0.91 mm (0.70–1.1 mm); male nymphs had a length of 3.5 mm (3.4–3.7 mm) and a width of 0.87 mm (0.75–1.0 mm). The mouth was positioned ventrally (Figure 2B). The genital pore of males measured between 24.5 and 26 µm in diameter in nymphs.

Two pairs of sharp-pointed hooks were present, and each hook had a small, dorsally located, sharp-pointed accessory piece (Figure 2C). Four pairs of sensory papillae were observed (Figure 2D): the first pair of sensory papillae was found on the antero-ventral margin of the body and was very difficult to observe; the second pair of papillae was observed lateral to the posterior pair of hooks around the second abdominal annulus; the third pair of papillae was found close to the midline of the ventral surface; and the fourth pair of sensory papillae was located on the midline of the fifth abdominal annulus. Female nymphs had approximately 87 (81–93) annular spines and males had approximately 81 (78–83) annular spines (Figure 2E). The structure of the sensory pores could be seen on each annulus (Figure 2E). The posterior side of female nymphs showed a terminal cleft 41–134 µm long (Figure 2F).

### 2.2. Molecular Analysis

The Agarose electrophoresis revealed specific bands at 1045 bp for the COX gene and 976 bp for the 18S rRNA gene. The sequences of the COX1 gene and 18S rRNA were submitted to GenBank and accessions received (OQ693945). The COX1 sequence showed 100% identity to MT196141.1, KF029447.1, MN481628 accessions of *L. serrata* from rabbit, dog and sheep, respectively.

### 2.3. Histopathology Results

**Figure 3:**
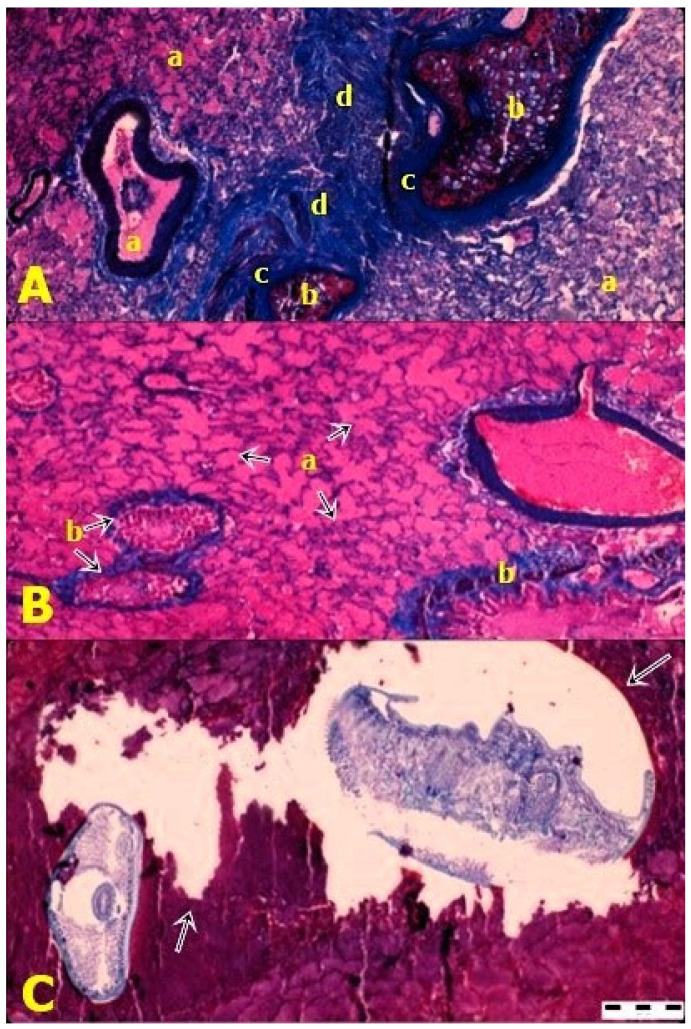
(**A**) a—Inflammatory pulmonary oedema; granulomas: b—central necrosis and calcification, c—fibrous tissue and d—predominantly eosinophilic leukocyte infiltrate; (**B**) a—Generalized pulmonary oedema and b—fibrosis of the bronchial and bronchiolar epitheliums; (**C**) Cyst containing the nymphal stage of *Linguatula serrata* (arrows).

Tissue preparations from the lung showed generalized inflammatory oedema (Figure 3A), fibrosis in the bronchial tree (Figure 3B), numerous parasitic granulomas (Figure 3A) and the presence of *L. serrata* nymphs (Figure 3C).

## 3. Discussion

This study presents the first report of a *L. serrata* in hares from Romania using morphological and molecular characterization tools.

In Romania, *Linguatula serrata* has been previously reported both in wild animals such as deer, foxes and jackals [35] and wolves [28,35] and in domestic animals such as dogs [26,27], but also in animals of neighbouring areas from Italy [36], the Balkans [37], and Greece [38,39]. Most reports of *L. serrata* nymphs in intermediate hosts reveal them encapsulated in a visible tissular capsule, such as those collected from bovine lymph nodes [2,40]. In this study, *L. serrata* nymphs were found encapsulated in the lung parenchyma of hares, while in Greece, nymphs were found in the gastrointestinal tract and liver, indicating that nymph development in leporids can occur throughout the body, with nymphs not localized to a single region/organ [39].

Parasite identification in hares is important and completes the wide and heterogeneous panel of reported intermediate hosts. The presence of this preadult stage in wild animals emphasizes the relation between transmission and predator–prey interactions [36]. The European brown hares are important prey for carnivores so they are a significant source of infection with this parasite. Hunting dogs that are fed with organs of hares can be infected with *L. serrata*. Similar to dogs, humans can also become infected with *L. serrata* nymphs if they ingest parasitized organs of intermediate hosts or if they come into contact with infected carnivores. In humans, nymphal stages have been identified in the upper respiratory tract [41,42], pharynx [43], eyeball [14], and liver [20]. Clinical manifestations vary depending on the location of the parasite, with sneezing, coughing, burning sensation in the nasopharyngeal mucosa, sore throat, loss of voice, vomiting, fatigue, profuse nasal and tear secretions and an itchy sensation in the nose, palate and throat. In the liver, *L. serrata* parasitism evolves asymptomatically, and in the eye, the *L. serrata* infection is manifested with redness of the eyes and face, pain and progressive loss of vision [14,20,41,43]. Infection is likewise considered a foodborne disease in the Middle East and Asian countries [41,44,45], as it is associated with the consumption of insufficiently cooked meat from infected domestic and wild herbivores, mainly camels and cattle, as these animals are an important source of food in Islamic countries [46].

To date, most identifications of pentastomes have been based almost exclusively on morphological characteristics, with few species subjected to molecular characterization [1,3,4,32,35,47]. Molecular identification of *L. serrata* in wildlife allows the establishment of links between pathogen transmission in the food chain. At the time of writing, molecular methods appear rather underappreciated, as suggested by the presence of only 61 sequences of the COX1 gene of *L. serrata* in the NCBI database, of which only two are associated with recorded cases in Europe [1]. Among the molecular methods employed, COX1 gene analysis remains essential in terms of detection, identification and characterization of this parasite [48].

A comparison was made regarding the morphology of nymphs collected during this study to that of specimens collected by Rezaei (2012), Mohanta (2017) and Barton (2020) [4,33,34]. Body length, width, and number of annular spines were similar for all specimens, indicating that morphological development is consistent for *L. serrata* nymphs, regardless of host species. However, there were some morphological differences between the nymphs collected in this study and those collected by Banaja (1983) [49] from goats in Saudi Arabia. In this study, a total of four pairs of sensory papillae were identified by scanning electron microscopy (SEM) compared to the three pairs of sensory papillae described by Banaja (1983) [49]. In addition, all of Banaja’s (1983) [49] specimens had their forelimbs “ventrally oriented”, whereas the specimens in this study had straight forelimbs. These morphological differences between Banaja’s (1983) [49] specimens and the specimens collected in this study may be due to different collection methods or geographical variations. Descriptions of *L. serrata* nymphs in the literature are very varied, with very few providing a detailed description. It is known that there are nine nymphal stages for the parasite in the body of its intermediate host [7,50], and it is possible that morphological differences are due to the description of the different nymphal stages of the parasite.

Histopathologically, numerous parasitic granulomas and localized fibrosis of the bronchial tree were observed in this study. A granulomatous inflammation and the presence of intermediate forms of the parasite were also reported by Hajipour et al. (2019) in a study conducted in Iran. They performed histopathological preparations from the lymph nodes of sheep [51]. Other authors have also described the presence of a congestive–hemorrhagic reaction and abundant hemosiderin deposits, along with severe oedema, foci of caseous necrosis and calcifications in mesenteric lymph nodes in sheep [52]. Most often, the lesions are accompanied by secondary infections of viral, bacterial or even fungal nature. Other authors have also reported the presence of abscesses [53]. In India, Gill et al. (1968) [54] observed very thickened bronchial vessels in hare due to hypertrophic changes in the medial layer of the vessel; also, the vessels showed subintimal proliferation of connective tissue elements. The bronchi showed hypertrophic changes in epithelial cells; thus, there was a pronounced hyperplasia of peribronchial lymphoid cells, and the pulmonary alveoli were collapsed [54]. In bovine livers, Moralez Muñoz et al. (2020) [55] described four types of granulomas. The first type is characterized by leukocytic hyperplasia, predominantly eosinophilic and a weak mesenchymal reaction. The second type is described by a decrease in the number of eosinophils and a stratified clustering of leukocytes (predominantly macrophages and lymphocytes), along with a peripheral organization of fibroblasts and collagen fibres. In type three granuloma, the formed fibrous tissue interferes with the leukocyte infiltrate and areas of mineralization. A central necrosis with leukocyte infiltrate and multiple foci of mineralization have been reported by researchers in type four granuloma [55]. In humans, nymphal stages have been identified both in the lung, under the appearance of nodules [24], and in the anterior chamber of the eyeball, along with a subluxation of the lens and secondary glaucoma [56].

In view of the veterinary and human medical importance of linguatulosis, further investigations are recommended in both domestic and wild carnivores and intermediate hosts present in Romania.

## 4. Materials and Methods

### 4.1. Parasites

**Figure 4:**
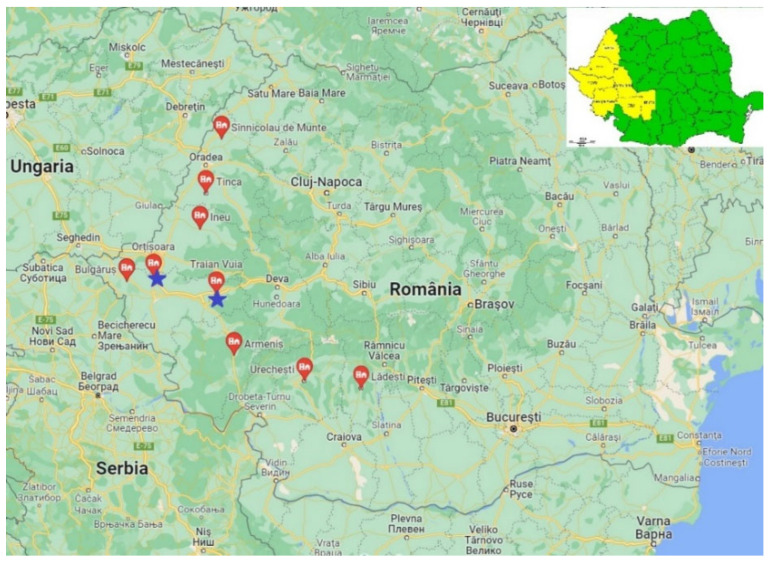
Map of the areas from which hares (*Lepus europaeus*) were collected (yellow colour). Geographical location of collected *Lepus europaeus* (red pin) infected with *Linguatula serrata* (blue star).

The study was carried out between November 2021 and February 2022 in nine localities from six counties located in the west and south-west of Romania. Hare carcasses pertaining to the hunting grounds within the study areas were collected from hunters (Figure 4, Table 2).

### 4.2. Morphological Examination

The hares were frozen in cool boxes at a temperature of 4 °C and transported to the Parasitology and Parasitic Diseases Laboratory of the Faculty of Veterinary Medicine, ULST for examination. Hares were necropsied and the heart, lungs, liver, spleen and mesenteric lymph nodes were removed and examined separately. Smaller organs were placed in a Petri dish with distilled water and examined under a microscope; the liver and lungs were sectioned into approximately 5 mm to 1 cm slices and examined.

After microscopic examination, parts of the liver and lungs were placed in different containers to which distilled water was added until the sample was covered with liquid. The tissues were then gently pressed to favor the removal of parasitic elements. The pressed tissues were washed, then removed, and the liquid was retained and poured through a 150 µm sieve into a Petri dish and examined under a microscope. All collected nymphs were immediately preserved in 70% ethanol, then fixed in lactophenol for morphological measurements (total body length and maximum width). After performing the measurements, two *L. serrata* nymphs were collected and placed into a 90% ethanol for molecular analysis.

### 4.3. Polymerase Chain Reaction

A small piece of the body of the *L. serrata* nymph was cut for DNA extraction.

Molecular identification was achieved by isolation, amplification, sequencing and analysis of the complete nuclear 18S rRNA gene and a portion of the mitochondrial cytochrome c oxidase subunit I gene (COX1).

Extraction of parasitic genomic DNA was performed using the Bioline-ISOLATE II Genomic DNA Kit (BIOLINE^®^, London, UK) employing the tissue protocol. The technique described by Gjerde (2013) [1] was used with minor modifications. Polymerase chain reaction (PCR) was conducted in 25 μL reactions using DNA, Master Mix My Taq TM Red Mix (BIOLINE^®^) and the specific primer pair for the identification of the COX1 gene: COX1LF (GCGACAATGACTATTTTCAACAAA) and COX1LR (GCGGTATCGATTGAGGAGTT).

For the amplification of the 18S rRNA gene, the primers used were ERIB1 (ACCTGGTTGATCCTGCCAG) and 3Hr (GGCAAATGCTTTCGCAGTAG) (Gjerde et al. 2013). Amplification was performed in the My Cycler (BioRad^®^, Hercules, CA, USA) thermocycler using the temperatures previously described (Gjerde 2013) adapted to the My Taq TM Red Mix (BIOLINE^®^).

PCR products were visualized on a 2% agarose using MidoriGreen fluorescent color (Nippon Genetics^®^ Europe, Düren, Germany).

PCR products were sequenced in the forward and reverse direction by Macrogen Europe B.V., Amsterdam, The Netherlands. Homology search was performed using NCBI BLAST.

### 4.4. Histopathology

Given the identification of *L. serrata* nymphs only in the lungs, lung tissue samples were taken and histopathological examination was carried out. Samples were preserved in a 70% ethanol and kept so for 24 h, after which they were clarified with xylene and embedded in 3 paraffin baths (1 h each at 55 °C). The paraffin blocks were processed using an ARM 3500 microtome. The resulting tissue fragments were deparaffinized with xylene (3 successive baths, 3 min each) and subsequently dehydrated with ethanol. Three concentrations of ethanol were employed: 96%, 80% and 70%. Each ethanol dip duration was 3 min. After the dehydration step, a two-minute hydration step was performed. Samples were further stained with 1% hematoxylin (5 min) and 1% eosin (2 min). After each staining, the samples were washed with distilled water. The samples were then immersed in a 1% phosphomolybdenic acid and a 1% methylene blue solution. During the final processing stages, samples were successively dehydrated with ethanol (70%, 80% and 90%) and clarified with xylene. Mounting of the samples was carried out with Canada balsam.

### 4.5. Scanning Electron Microscopy (SEM)

One nymph collected during the study was selected for electron microscopic scanning. The nymph was washed in an anhydrous ethanol solution, then dried with liquid CO_2_. The sample was placed ventral side up on a carbon wire holder attached to a SEM pin mount and coated with gold ions for 2 min at 15 mA in a Quorom SC7620 sputter. The Hitachi TM3000 microscope was used to capture the images.

## 5. Conclusions

This study presents the first report on *Linguatula serrata* nymphs collected from hares in Romania using both molecular and morphological characterization. The study demonstrates that the European brown hare is a reservoir for immature forms of *L. serrata* and highlights the importance of veterinary evaluation of hare carcasses for the identification of this parasite. Morphological features revealed via using the SEM technique show the life cycle stage of the nymph and prove to be a useful tool in the morphological characterization of evolutionary stages. *Linguatula serrata* nymphs in hares from Romania were identified in the lungs where they caused the following lesions: generalized inflammatory oedema, granulomas with necrosis, calcifications and fibrosis in the bronchial tree. Molecular identification and similarity of the isolates to other isolates from hare and carnivores highlight the importance of trophic links in the spread of this zoonotic disease. The sequence obtained from the molecular analysis has been deposited in GenBank under accession number OQ693945.

## Figures and Tables

**Table 1:** Morphometry of *Linguatula serrata* nymphs.

Measured Region		Present Study			Barton et al. (2020) [4]	Mohanta et al. (2017) [33]	Rezaei et al. (2012) [34]
		Minimum	Maximum	Average			
**Female nymph body (mm)**	Length	3.0	5.5	4.2	3.7	4.2	4.9
	Width	0.7	1.1	0.91	0.89	1.2	1.0
**Male nymph body (mm)**	Length	3.4	3.7	3.5	3.5	NA	NA
	Width	0.75	1.0	0.87	0.76		
**No. of annular spines**	female nymphs	81	93	87	83	88	NA
	male nymphs	78	83	81	82	NA	NA
**Genital pore of male nymphs (µm)**		24.5	26	NA	NA	NA	NA
**Posterior part of female nymphs (terminal cleft) (µm)**		41	134	NA	NA	NA	NA

Note: NA—not available.

**Table 2:** Distribution of *Lepus europaeus* infected with *Linguatula serrata*.

Location	Number of *L. europaeus* Studied	Number of *L. europaeus* Infected
**Timiș County**	9	2
**Arad County**	5	-
**Bihor County**	3	-
**Caraș-Severin County**	2	-
**Gorj County**	3	-
**Vâlcea County**	2	-
**TOTAL**	**24**	**2**

## Data Availability

All the datasets generated or analyzed during this study are included in this published article.
